# Whole Heart Dose Parameters Predict Severe Arrhythmias After Neoadjuvant Chemoradiotherapy for Esophageal Squamous Cell Cancer: A Competing Risk Analysis of 358 Patients

**DOI:** 10.1002/cam4.71610

**Published:** 2026-02-08

**Authors:** Wei‐Xiang Qi, Haiping Zhang, Xunmei Yang, Shuyan Li, Mengdi Wang, Biao Yu, Linlin Chen, Gang Cai, Cheng Xu, Jiayi Chen, Shengguang Zhao

**Affiliations:** ^1^ Department of Radiation Oncology, Ruijin Hospital Shanghai Jiaotong University School of Medicine Shanghai China; ^2^ Shanghai Key Laboratory of Proton‐Therapy Shanghai China; ^3^ Department of Medical Oncology Linfen People's Hospital Linfen China; ^4^ Department of Oncology People's Hospital of Qiandongnan Miao and Dong Autonomous Prefecture Kaili China

**Keywords:** arrhythmia, cardiac toxicities, esophageal cancer, neoadjuvant chemoradiotherapy, supraventricular cardiac conduction system

## Abstract

**Background:**

Dose exposure to supraventricular cardiac conduction system doses has been reported to be associated with distinct arrhythmia classes after thoracic radiotherapy, but its impact in esophageal squamous cell carcinoma (ESCC) remains unknown.

**Materials and Methods:**

Locally advanced ESCC treated with neoadjuvant chemoradiotherapy (nCRT) were included. The primary endpoint was grade ≥ 3 adverse cardiac arrhythmia events. Prediction performance was evaluated through time‐dependent receiver operating characteristic curves, and competing risk frameworks were implemented to quantify the cumulative incidence of distinct cardiac arrhythmia.

**Results:**

Of 358 patients, 84.9% were men, with a median age of 66 years (range: 39–79 years). A total of 60 (16.8%) patients experienced at least 1 grade ≥ 3 arrhythmia, with a median time to first arrhythmia of 13 months (95% CI: 12–15 months). The 2‐year cumulative incidences of distinct cardiac arrhythmia were 8.89% for AF, 2.96% for atrial flutter, and 5.12% for other SVT. Baseline coronary heart disease was a risk factor for all types of arrhythmia (*p* < 0.05). After adjusting for baseline cardiovascular risk factors, Heart *D*_max_ (sHR 3.69, *p* = 0.0024) was associated with AF, Heart volume receiving 5 Gy with atrial flutter (sHR: 9.35, *p* = 0.0077), and Heart volume receiving 40 Gy (sHR 5.72, *p* = 0.00078) with other SVT.

**Conclusion:**

Grade ≥ 3 cardiac arrhythmia associated with thoracic radiation occurs in 16.7% of ESCC patients undergoing nCRT within a median time of 13 months. The radiation dose exposure of the supraventricular cardiac conduction system is not associated with increased cardiac arrhythmia. Specific arrhythmia subtypes exhibited differential associations with distinct dose‐volume parameters of whole heart irradiation.

## 1 Introduction

Esophageal cancer is one of the most common cancers in China and caused a huge disease burden, with an estimated of 184,500 incident cases of esophageal cancer and 142,300 deaths in China [1]. Esophageal squamous cell carcinoma (ESCC) was the most common histological type in China, accounting for 85.79% of all cases. Currently, neoadjuvant concurrent chemoradiation followed by esophagectomy became the standard of care for locally advanced esophageal cancer patients based on promising results of the phase 3 CROSS trial [2] and NEOCRTEC5010 trial [3]. However, Emerging evidence from population‐based cohorts reveals a pathogenic cascade wherein radiotherapy‐induced AF acts as a prognostic mediator, exacerbating disease progression in esophageal cancer. Song et al. [4] found that older patients or patients receiving higher radiation dose were two independent risk factors for developing AF after surgery. While Byer et al. [5] noted that the development of new‐onset AF after esophagectomy was associated with significantly worse survival (HR = 1.56, 95% CI: 1.21–2.02, *p* < 0.001). Similarly, Miller E.D also showed that radiation therapy increases AF risk for esophageal cancer patients, and patients developed AF were associated with worse long‐term outcomes [6]. However, the treatment regimen for esophageal cancer in those published studies had substantial heterogeneity, encompassing both neoadjuvant or curative RT, with marked divergence in concurrent chemotherapy regimens. All of these factors would impact the incidence rate of arrhythmia in this patient population.

Additionally, there remains a paucity of data that thoroughly characterizes distinct categories of arrhythmias with dose‐volume predictors linked to cardiac conduction. On the other hand, the sinoatrial node (SAN) functions as the physiological pacemaker of the heart, situated in the right atrium at the junction between the crista terminalis and the superior vena cava. Iovoli et al. [7] reported that SAN *D*_max_ and *D*_mean_ were significantly associated with worse overall survival among central lung cancer patients undergoing SBRT. Kim et al. found that incidental irradiation of the SAN during nCRT for lung cancer was associated with the development of AF and increased mortality [8]. These published data supported regarding cardiac conduction system as a new organ at risk in thoracic radiotherapy. However, the irradiation of cardiac conduction system on the occurrence of arrhythmia in esophageal cancer remained undetermined. As a result, the aim of the present study aimed to comprehensively assess the incidence of distinct classes of arrhythmias in ESCC patients treated with standardized nCRT, and investigate the influence of radiation exposure to cardiac conduction nodes (SAN and AVN) and whole heart on the risk of developing cardiac arrhythmia.

## 2 Material and Methods

### 2.1 Study Population

This investigation was conducted utilizing a prospectively maintained institutional database (ClinicalTrials.gov identifiers: NCT03792347, NCT04435197, NCT04513418, NCT03990532, NCT06596954). The study population comprised consecutive patients who received standardized neoadjuvant chemoradiotherapy (nCRT) followed by curative resection from January 2019 to December 2024. The comprehensive eligibility criteria for these prospective cohorts have been previously delineated in our protocol publications [9, 10, 11].

The major eligibility criteria included: (1) age 18–75 years; (2) radiologically confirmed locally advanced esophageal squamous cell carcinoma (ESCC; cT3‐4 and/or N+); (3) treated with standardized neoadjuvant chemoradiotherapy (nCRT) comprising weekly concurrent carboplatin (AUC = 2) with paclitaxel or nab‐paclitaxel (50 mg/m^2^), followed by radiotherapy delivering 41.4 Gy in 23 fractions. For patients enrolled in the PALACE trial, two additional cycles of pembrolizumab (200 mg) were administered on Days 1 and 22 during neoadjuvant therapy [11]. Detailed methodologies for target volume delineation—including gross tumor volume (GTV), clinical target volume (CTV), and planning treatment volume (PTV) – have been comprehensively described in our prior publications [9, 12, 13]. Planning was performed using 6–10 MV energy with intensity‐modulated radiation therapy (IMRT) or volume Modulated Arc Therapy (VMRT).

### 2.2 Potential Clinical Predictors

Candidate predictors for statistical modeling were identified through multidisciplinary consensus and published evidence [14]: encompassing: (1) demographic parameters (age, sex, body mass index); (2) tumor characteristics (clinical stage, longitudinal tumor extent); (3) treatment modality (neoadjuvant chemoradiotherapy [nCRT] ± pembrolizumab); (4) pre‐existing arrhythmia; (5) comorbidities including diabetes mellitus, hypertension, coronary heart disease (CHD), chronic obstructive pulmonary disease (COPD), cerebrovascular accident, gout, asthma, and pulmonary embolism; (6) behavioral factors (tobacco use, alcohol consumption).

CHD was operationally defined as: (a) documented coronary artery disease, (b) ischemic cardiomyopathy, or (c) established CHD risk equivalents (peripheral artery disease or prior ischemic stroke).

### 2.3 Primary Endpoint

The primary endpoint of the present study was grade ≥ 3 arrhythmia events classified according to Common Terminology Criteria for Adverse Events (CTCAE v4.0), systematically adjudicated through a structured electronic medical record (EMR) abstraction protocol. This multisource verification integrated electrocardiographic reports, cardiology consultation notes, and arrhythmia‐specific medication records. Events were categorized as: (1) Atrial fibrillation (AF); (2) Atrial flutter; (3) Other supraventricular tachyarrhythmias (SVT), encompassing sustained supraventricular tachycardia, symptomatic sinus tachycardia (> 120 bpm at rest), and unspecified atrial arrhythmias. Routine 12‐lead ECG at baseline, during nCRT, preoperatively, and at each follow‐up visit was performed. And 24‐h Holter monitoring for symptomatic patients or those with abnormal ECG findings. The timing of monitoring was consistent across the cohort, with follow‐up visits scheduled at 3‐month intervals for the first 2 years.

### 2.4 Cardiac Structures Delineation and Dosimetry

According to the contouring atlas [15], the SAN and AVN were contoured in manually by X.Y. and H.Z. In brief, the sinoatrial node (SAN) was delineated along the crista terminalis of the right atrium, extending between the superior and inferior venae cavae. Anatomically, it was represented as a 2‐cm‐diameter spherical volume (tangent to the right atrial epicardium) centered at the craniocaudal level corresponding to the origin of the ascending aorta. The atrioventricular node (AVN) was localized within Koch's triangle, bounded by the tricuspid valve septal leaflet, coronary sinus ostium, and the tendon of Todaro. To account for anatomical variability and imaging resolution limitations, the AVN was approximated as a 2‐cm‐diameter sphere centered on the atrioventricular septal junction (intersection of all four cardiac chambers), positioned 1 cm superior to the most cranial CT slice demonstrating left atrial visibility. Once the SAN and AVN were contoured, the dose maximum (*D*_max_), dose mean (*D*_mean_), V5, V10, V15, V20, V25, V30, V35 and V40 for each structure and whole heart were calculated.

### 2.5 Statistical Analysis

Follow‐up duration was determined from the initiation of nCRT via the reverse Kaplan–Meier approach. Continuous variables are expressed as medians with interquartile ranges (IQRs; Q1–Q3), while categorical variables are reported as frequencies and percentages. Cumulative incidence rates of arrhythmia subtypes—specifically grade ≥ 3 atrial fibrillation (AF), atrial flutter, and non‐AF/non‐atrial flutter supraventricular tachycardia (other SVT)—were estimated with 95% confidence intervals (CIs), incorporating noncardiac death as a competing risk. Comparisons between groups were conducted using Gray's test. Fine‐Gray proportional hazards regression models were applied to account for competing risks from noncardiac mortality, with outcomes reported as subdistribution hazard ratios (sHRs) and 95% CIs. Due to the high multicollinearity among different dose‐volume histogram parameters, we adopted a two‐step approach to identify dosimetric predictors independently associated with the outcomes. First, we performed time‐dependent receiver operating characteristic (TimeROC) curve analysis to rank each candidate dosimetric parameter based on the area under the curve and selected the top five parameters for each type of arrhythmia. Second, to avoid collinearity, each selected dosimetric parameter was individually incorporated into a multivariable Fine‐Gray proportional hazards model, which simultaneously adjusted for all predetermined clinicopathological factors (e.g., age, baseline coronary heart disease). The adjusted hazard ratios from these models are reported. To internally validate our findings and assess the robustness of variable selection, we performed bootstrap resampling with 500 iterations. In each iteration, the entire modeling procedure—comprising univariable screening via Fine‐Gray regression (*p* < 0.1), followed by multivariable model construction—was repeated. This process allowed us to calculate the frequency with which each candidate dosimetric predictor was selected across all bootstrap samples. All analyses were conducted using R statistical software (version 4.4.2).

## 3 Results

### 3.1 Baseline Characteristics

Baseline clinical and treatment characteristics were summarized in Table 1. The median age was 66 years (Q1–Q3: 60–70 years), and 84.9% (*n* = 304) were men. 78.8% of patients (*n* = 282) with BMI > 20. The majority of stage were III stage (94.2%, *n* = 337) with median tumor length of 5 cm. all included patients were squamous cell carcinoma and treated with standardized nCRT regimen (RT does: 41.4 Gy/23 Fx), and 84.9% of them (*n* = 301) received curative surgery after nCRT. As for comorbidities, 21.2% (*n* = 76), had pre‐existing arrhythmia diagnoses prior to nCRT, and other common cardiac comorbidities included hypertension in 35.5% (*n* = 127), hyperlipidemia in 12.6% (*n* = 45), diabetes mellitus in 9.8% (*n* = 35), any coronary heart disease in 8.4% (*n* = 30), prior stroke in 4.2% (*n* = 15). The dosimetric parameters were listed Table S1. The median *D*_max_ and *D*_mean_ for whole heart was 4444.05 cGy and 1545.05 cGy, respectively. The median low to high dose exposure to heart was 80.01% for V5, 58.84% for V10, 41.08% for V15, 29.94% for V20, 20.58% for V25, 13.44% for V30, 9.28% for V35 and 5.17% for V40 respectively. While the median to high dose exposure to SAN and AVN was very low, the median of V30, V35 and V40 for SAN and AVN was 0%. The median *D*_max_ and *D*_mean_ dose for SAN and AVN was 2617.4 cGy vs. 2749.3 cGy and 2003.35 cGy vs. 1845.65 cGy, respectively.

**TABLE 1:** Baseline characteristics of included patients.

Characteristics	*N* (%)
Age at diagnosis	
Median (Q1–Q3), years	66 (60–70 years)
< 65 years	152 (42.5%)
≥ 65 years	206 (57.5%)
BMI (kg/m^2^)	
≤ 20	76 (21.2%)
> 20	282 (78.8%)
Sex	
Female	54 (15.1%)
Male	304 (84.9%)
Stage	
II	7 (2.0%)
IIIA	16 (4.5%)
IIIB	321 (89.7%)
IVA	14 (3.9%)
Tumor length, cm	Median 5 cm, range (1–17 cm)
Comorbidities	
Hypertension	127 (35.5%)
Arrhythmia	76 (21.2%)
Hyperlipidemia	45 (12.6%)
CHD	30 (8.4%)
Diabetes mellitus	35 (9.8%)
Stroke	15 (4.2%)
Gout	9 (2.5%)
Asthma	2 (0.6%)
COPD	3 (0.8%)
Pulmonary embolism	2 (0.6%)
Drinking status, *n* (%)	
Never	128 (35.8%)
Current/former	230 (64.2%)
Smoking status, *n* (%)	
Never	128 (35.8%)
Current/former	230 (64.2%)
Tumor histology, *n* (%)	
Squamous cell carcinoma	358 (100%)
Prescribed RT dose, Gy	41.4 Gy/23 Fx
Neoadjuvant chemotherapy regimen	
Paclitaxel + carboplation	30 (8.4%)
Nab‐paclitaxel + carboplation	325 (91.6%)
Curative surgery after *n*CRT	
No	57 (15.9%)
Yes	301 (84.1%)
nCRT regimen	
With pembrolizumab	129 (36.0%)
Without pembrolizumab	229 (64.0%)

### 3.2 Arrhythmia Events

**FIGURE 1:**
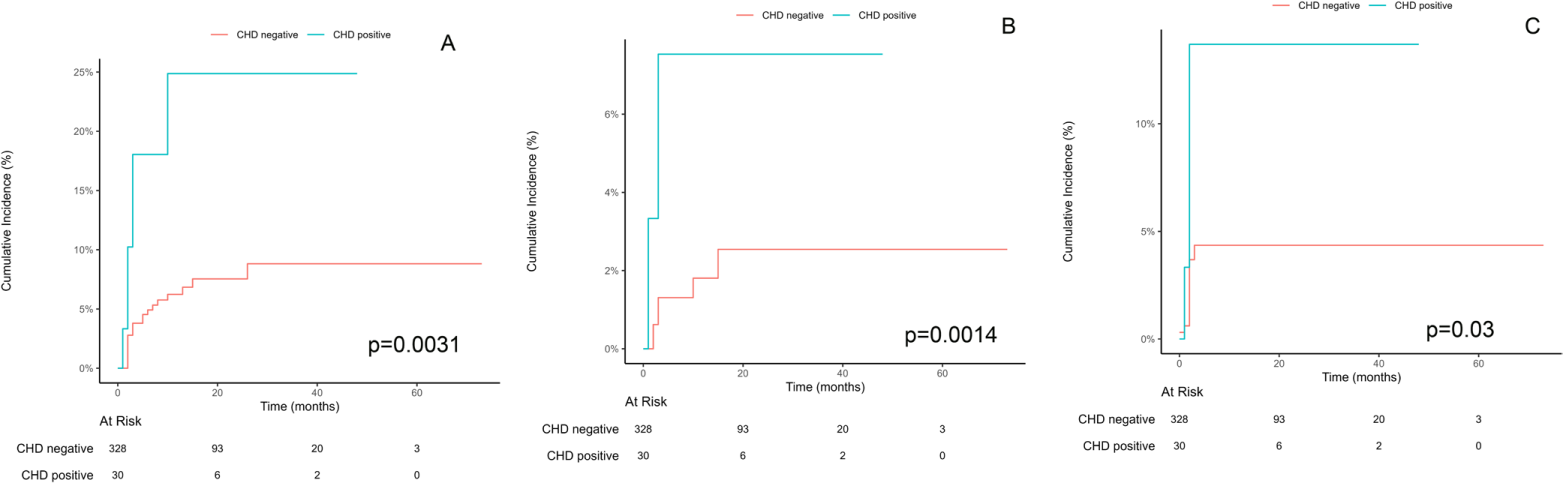
Cumulative incidence of specific cardiac arrhythmia associated according to baseline coronary heart disease: (A) Atrial fibrillation; (B) atrial flutter; (C) Non‐AF/non‐atrial flutter supraventricular tachycardia.

**FIGURE 2:**
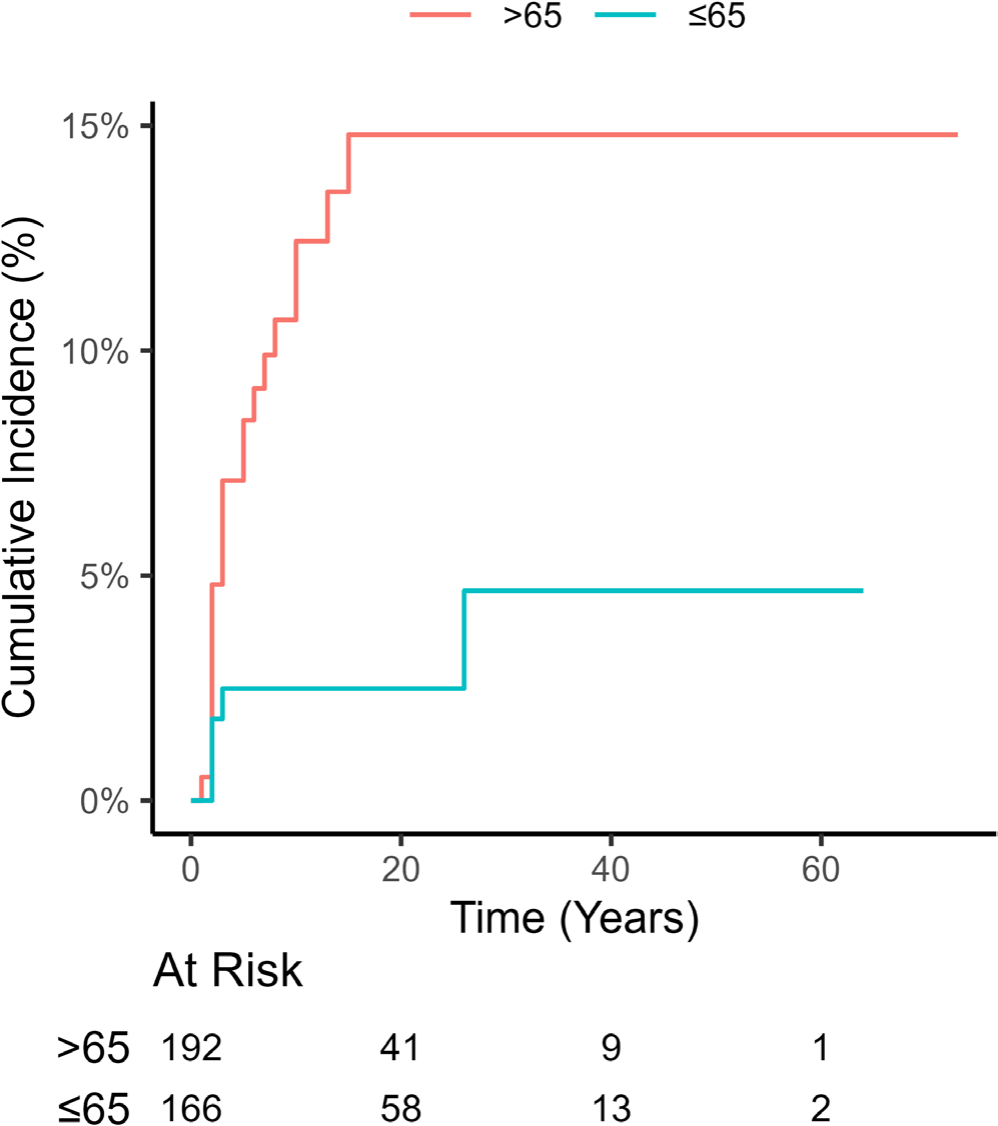
Cumulative incidence of atrial fibrillation according to patient age.

Of 358 patients, 60 (16.8%) experienced at least 1 grade ≥ 3 arrhythmia, with a 2‐year overall cumulative incidence of 11.9% (95% CI: 9.7%–14.4%) and a median time to first arrhythmia of 13 months (95% CI: 12–15 months). A total of 13 patients died from esophageal cancer, with a 2‐year cumulative incidence of 3.9%. The 2‐year cumulative incidence of AF was 8.89%, with a median time to first AF of 12 months (95% CI: 11–14 months). The 2‐year cumulative incidence of atrial flutter was 8.89%, with a median time to first AF of 11 months (95% CI: 10–13 months). The 2‐year cumulative incidence of other SVT was 5.12%, with a median time to first AF of 12 months (95% CI: 11–13 months). Patients who had a history of coronary heart disease were more likely to develop grade ≥ 3 AF (24.9% vs. 7.5%), atrial flutter (7.5% vs. 2.5%), and other SVT (13.6% vs. 4.35%, Figure 1). In addition, patients of elder age (14.8% vs. 2.49%, Figure 2) or pre‐existing hypertension (15.4% vs. 5.5%) were more likely to develop grade ≥ 3 AF, but the development of atrial flutter or other SVT was not associated with age and baseline hypertension.

### 3.3 Dose Predictors of Distinct Arrhythmia Groups

The top 5 dose‐volume histogram predictors by area under the curve for each arrhythmia group were shown in Table 2. An association between AF with moderate to high dose exposure to the whole heart, atrial flutter with low to moderate dose exposure to the heart and AVN, and other SVT with moderate to high dose exposure to the heart and AVN; no association between arrhythmia and dose exposure to SAN was observed. The areas under the curve for all dose‐volume histogram parameters and substructures were detailed in Table S2.

**TABLE 2:** ROC of the top 5 dose volume parameters for cardiac substructures predictive for atrial fibrillation, atrial flutter, and other VAT in ESCC cohort.

Predictors for AF	ROC value	Predictors for atrial flutter	ROC value	Predictors for other VAT	ROC value
Heart *D*_max_	0.602	Heart V5	0.6424	Heart V30	0.6779
Heart *D*_mean_	0.592	Heart V30	0.5841	Heart V35	0.6783
Heart V5	0.5651	Heart V40	0.5826	Heart V40	0.6704
Heart V10	0.5658	AVN V20	0.5876	AVN *D*_mean_	0.6606
Heart V40	0.6107	AVN V40	0.6174	AVN V25	0.6678

**FIGURE 3:**
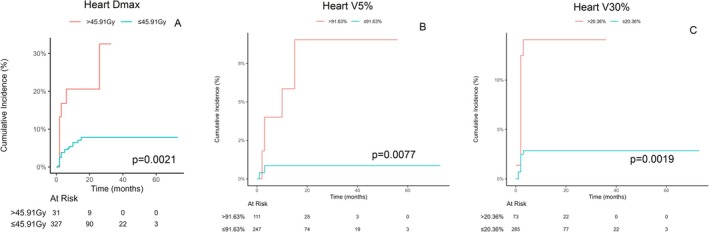
Specific cardiac arrhythmia associated according to heart radiation exposure: (A) Cumulative incidence of atrial fibrillation according to heart *D*_max_; (B) cumulative incidence of atrial flutter according to Heart V5; (C) Cumulative incidence of non‐AF/non‐atrial flutter supraventricular tachycardia according to heart V30.

On competing risk regression, adjusting for age, baseline hypertension or coronary heart disease, heart *D*_max_ > 45.91 Gy was associated with an increased risk for AF (sHR 3.69, 95% CI: 1.59–8.56, *p* = 0.0024, Figure 3A and Table 3). Adjusting for pre‐existing coronary heart disease, heart V5 > 91.63% was associated with an increased risk for atrial flutter (sHR: 9.35, 95% CI: 1.8–48.4; *p* = 0.0077, Figure 3B and Table S3). Adjusting for pre‐existing coronary heart disease, heart V30 > 20.36% was associated with an increased risk for other SVT (sHR: 4.65; 95% CI: 1.77–12.22; *p* = 0.0019, Figure 3C and Table S4).

**TABLE 3:** Competing risk regression models for Atrial Fibrillation.

Characteristics	Univariate		Multivariate	
	HR (95% CI)	*p*	sHR (95% CI)	*p*
Age				
≤ 65	1		1	
> 65	4.18 (1.6–11)	0.0036	1.34 (1.49–9.80)^a^	0.0054
Sex				
Female	1			
Male	1.09 (0.38–3.12)	0.87		
BMI				
< 20	1			
≥ 20	0.55 (0.25–1.19)	0.13		
Baseline hypertension				
No	1		1	
Yes	2.42 (1.14–5.14)	0.021	0.54 (0.81–3.59)^a^	0.16
Diabetes				
No	1			
Yes	2.12 (0.81–5.55)	0.13		
Baseline arrhythmia				
No	1			
Yes	0.82 (0.32–2.14)	0.69		
Baseline CHD				
No	1		1	
Yes	3.84 (1.58–9.37)	0.0031	3.18 (1.38–7.31)^a^	0.0066
Tumor length	0.97 (0.83–1.14)	0.73		
Surgery				
No	1			
Yes	4.52 (0.62–33)	0.14		
nCRT Regimen				
Without pembrolizumab	1			
With pembrolizumab	0.60 (0.26–1.4)	0.24		
Smoking status				
No	1			
Yes	0.85 (0.40–1.8)	0.67		
Drinking status				
No	1			
Yes	0.72 (0.34–1.52)	0.39		
RT dose				
Heart *D*_max_ (≤ 45.91 Gy vs. > 45.91 Gy)	3.76 (1.61–8.75)	0.0021	3.69 (1.59–8.56)^a^	0.0024
Heart V5, mL (≤ 84.4% vs. > 84.4%)	3.2 (1.45–7.07)	0.0041		
Heart V30, mL (≤ 21.38% vs. > 21.38%)	2.17 (0.96–4.88)	0.062		
Heart V35, mL (≤ 14.59% vs. > 14.59%)	2.05 (0.91–4.63)	0.082		
Heart V40, mL (≤ 4.13% vs. > 4.13%)	2.33 (0.94–5.79)	0.069		

Abbreviation: CHD, coronary heart disease.

^a^ Multivariate analysis with age, baseline Hypertension, baseline CHD, and heart *D*_max_.

The bootstrap resampling procedure identified the most robust predictors based on their selection frequency across 500 iterations. For the predictors of AF, clinical variables predominated, with age (selected in 74% of samples) and CHD (64%) exhibiting the highest stability. Regarding dosimetric parameters, the maximum dose to any structure (*D*_max_, 54%) demonstrated the greatest robustness. For the dosimetric predictors of atrial flutter, V5 (60%) and V30 (46%) were frequently retained. For the dosimetric predictors of other SVT, V30 (84%), V35 (75%), and V40 (77%) were frequently retained.

The bootstrap resampling (500 iterations) revealed distinct predictor profiles for each arrhythmia type. For atrial fibrillation (AF), clinical variables—specifically age (74%) and CHD (64%)—were the most robust predictors, alongside *D*_max_ (47%). In contrast, the risk of atrial flutter was best predicted by the volume of heart receiving low to moderate doses, with V5 (56.2%), V10 (44.8%), and V30 (45.6%) being most stable. For other supraventricular tachycardias (SVT), high dose volumes to the heart demonstrated the greatest predictive robustness, evidenced by the high selection frequencies of V30 (84%), V35 (75%), and V40 (70%).

## 4 Discussion

Prior studies on radiation‐associated arrhythmias in esophageal cancer had largely focused on definitive chemoradiotherapy (CCRT) or radiotherapy alone cohorts. For instance, Miller et al. [6] demonstrated in a mixed cohort of esophageal cancer patients (including both definitive and adjuvant settings) that 21.4% of 238 patients developed incident AF after radiotherapy. In contrast, Butler et al. [16] reported that the 5‐year cumulative incidence of AF was 8.3% for esophageal cancer after definitive radiotherapy. Additionally, studies focusing on lung cancer populations, such as those by Atkins et al. [14] and other two studies [17, 18] reported lower incidences of new‐onset AF (6%–11.2%). Since the publication of CROSS trial [2] and NEOCRTEC5010 trial [3], nCRT followed by surgery were the standard of care for locally advanced ESCC. It was clearly needed to provide detailed dosimetric and arrhythmia adjudication data for this patient population, which were often lacking in nCRT cohorts. In our study, the 2‐year cumulative incidences of distinct cardiac arrhythmia were 8.89% for AF, 2.96% for atrial flutter, and 5.12% for other SVT, which are more aligned with the rates reported by Atkins et al. in lung cancer [14] but significantly lower than those reported by Miller et al. in a mixed esophageal cancer cohort [6]. This discrepancy might be attributable to several factors: the median radiation dose in Miller et al.'s study was 50.4 Gy, and there was substantial heterogeneity in concurrent chemotherapy regimens. In contrast, our study employed a standardized nCRT regimen (41.4 Gy with paclitaxel/nab‐paclitaxel and carboplatin). Previous studies had suggested that fluoropyrimidine‐based regimens, as opposed to carboplatin/paclitaxel, might be associated with an increased risk of AF [19, 20], which could partly explain the lower incidence observed in our cohort. Additionally, we found that patients with a history of coronary heart disease or advanced age were more likely to develop AF, while atrial flutter and other SVT were significantly associated with baseline coronary heart disease, consistent with previous findings [21].

The pathophysiology of AF is diverse and has not been fully elucidated. Sinus node dysfunction (SND) frequently coexists with SAN dysfunction, which is considered as a predisposing condition for AF. Indeed, there is a growing evidence data demonstrating strong associations between AF and mortality following thoracic radiotherapy. AF is independently associated with an increased risk of incident myocardial infarction and stroke [22]. Two previous studies indicated that esophageal cancer patient presented with new‐onset AF were associated with worse long‐term outcomes [5, 6]. A recent systematic review also showed that higher doses to the conduction system, especially the SAN, were associated with a higher incidence of a wide range of arrhythmias and poorer overall survival [23]. As a result, it was important to clearly determine the association between dose to cardiac substructures and AF after thoracic radiotherapy. Indeed, several studies had been published to investigate the association between the dose to cardiac substructures and AF among cancer patients. In a recent study, left atrium (LA) dose was associated with increased risk of incident AF among esophageal cancer [18], while Walls reported that radiation dose to the pulmonary veins during treatment for NSCLC was associated with the onset of AF [17]. However, the underlying mechanisms of atrial fibrillation differed significantly from those of acute coronary syndromes or heart failure, necessitating evaluation of cardiac components beyond the ventricular chambers and coronary vasculature. Therefore, Kim et al. [8] firstly demonstrated that the maximum dose (*D*_max_) to SAN best predicted new AF from the available cardiac substructure dose‐volume histogram (DVH) metrics in a cohort of 321 patients with NSCLC. In the present study, we investigated the association of supraventricular cardiac conduction system and risk of specific cardiac arrhythmia. However, our result showed that there was no significant association between cardiac arrhythmia and dose to SAN/AVN. One possible explanation for this finding was that the dose exposure to SAN in the present study was lower than those reported in lung cancer patients. After adjusting baseline cardiovascular risk factors, Heart *D*_max_ remained associated with increased risk of AF, heart volume receiving 5 Gy with atrial flutter, Heart volume receiving 40 Gy with other SVT. Our findings challenged the conventional emphasis on avoiding conduction system irradiation in esophageal cancer RT planning. Instead, a heart‐sparing approach targeting global dose reduction might yield greater clinical benefits.

A related issue that must be acknowledged is the potential overlap between surgery‐induced and radiation‐associated arrhythmias. As this study focused on arrhythmias after nCRT, the potential confounding effect of surgery‐induced AF is crucial to address. Postoperative AF (POAF) was a well‐documented complication after esophagectomy, with the majority of episodes occurring within the first 2 to 7 postoperative days. For example, Stawicki et al. reported an incidence of new‐onset AF in 20.5% (32/156) of patients after esophagectomy, with the majority of cases (87.5%) occurring within the first 72 h post‐surgery [24]. In the present cohort, the median time to the first grade ≥ 3 arrhythmia was 13 months, and the median time to the first AF event was 12 months. This temporal pattern was substantially later than the typical onset window for POAF, strongly suggesting that the arrhythmic events captured in our medium‐term follow‐up were distinct from acute postoperative complications and were more likely attributable to the late effects of radiotherapy. Although it was conceivable that some patients with subclinical POAF might have experienced late recurrences, the significant associations we observed between arrhythmia incidence and specific heart dosimetric parameters, even after adjusting for clinical risk factors, further reinforced the primary role of radiation exposure.

The major strength of this study was that our study was the largest prospective cohort of individual patient cardiac dosimetry in neoadjuvant thoracic radiotherapy with manual delineation of supraventricular cardiac conduction system (SAN and AVN) and to comprehensively assess the incidence of distinct classes of arrhythmias in ESCC patients. As a result, all enrolled patients treated with 41.4 Gy/23 Fx radiotherapy and concurrent chemotherapy (paclitaxel/nab‐paclitaxel and carboplatin), the incidence of arrhythmias could be easily externally validated in other institutions. However, the present study had the following limitations. First of all, we only investigated the association between radiotherapy dose exposure to supraventricular cardiac conduction system/whole heart and risk of arrhythmia; other cardiac substructures, such as pulmonary vein and cardiac chambers, were not assessed because detailed analysis of the cardiac substructure dose variables and their association with arrhythmia was outside of the scope of our study. Secondly, the long‐term survival outcomes were not available due to limited follow‐up time; thus, the impact of arrhythmia on the survival of ESCC was unknown. Finally, this was a retrospective study, and there was a lack of a validation cohort. Prospective studies investigating the association between cardiac substructure dose variables and cardiac arrhythmia were recommended. In addition, aggressive heart dose reduction might inadvertently increase lung exposure, potentially elevating the risk of radiation pneumonitis. Therefore, we emphasized the importance of individualized planning and the use of advanced techniques including proton radiotherapy or deep inspiration breath hold (DIBH) to balance these competing risks. Future studies should optimize multi‐objective planning to minimize both cardiac and pulmonary toxicity.

## 5 Conclusion

In conclusion, the overall incidence of grade ≥ 3 cardiac arrhythmia associated with thoracic radiation was 16.7% in ESCC patients undergoing standardized nCRT within a median time of 13 months. The radiation dose exposure of supraventricular cardiac conduction system was not associated with increased cardiac arrhythmia. Distinct arrhythmia classes were associated with radiotherapy dose to different whole heart dose exposures.

## Author Contributions

**Wei‐Xiang Qi:** conceptualization, writing – original draft, funding acquisition, formal analysis, data curation. **Haiping Zhang:** methodology, validation, data curation, supervision, resources. **Xunmei Yang:** investigation, validation, software, formal analysis, data curation. **Shuyan Li:** methodology, software, formal analysis, data curation. **Mengdi Wang:** validation, software, formal analysis, methodology, data curation, resources. **Biao Yu:** methodology, validation, supervision, software. **Linlin Chen:** validation, software, formal analysis, data curation. **Gang Cai:** project administration, data curation, conceptualization, writing – review and editing. **Cheng Xu:** investigation, software, project administration, supervision. **Jiayi Chen:** writing – review and editing, conceptualization, funding acquisition, software, supervision, project administration. **Shengguang Zhao:** writing – original draft, project administration, supervision, conceptualization, software.

## Funding

This study was supported in part by Shanghai Science and Technology Innovation Action Plan (grant number 23Y41900100), the National Key Research and Development Program of China (grant number 2022YFC2404602), Clinical Research Special Project of Shanghai Municipal Health Commission Health Industry (202340226), and Shanghai Science and Technology Innovation Action Plan Medical Innovation Research Project (23Y11904700). The funding agency plays no role in the design or execution of the study.

## Ethics Statement

This study was approved by the Ethics Committee of Ruijin hospital, Shanghai Jiao Tong University School of Medicine. The study was conducted in accordance with the Declaration of Helsinki. This study was a retrospective collection of prospective cohort data, and according to the ethical requirements of our center, it was not necessary to obtain informed consent from patients.

## Conflicts of Interest

The authors declare no conflicts of interest.

## Supporting information


**Table S1:** Dosimetric parameters for heart and conduction nodes.

**Table S2:** AUC value of 3‐year TimeROC result for arrhythmia.

**Table S3:** Competing risk regression models for Atrial Flutter.

**Table S4:** Competing risk regression models for other supraventricular tachycardia.

## Data Availability

The data that support the findings of this study are available from the corresponding author upon reasonable request.
