# Supplementary material for: Whole Heart Dose Parameters Predict Severe Arrhythmias After Neoadjuvant Chemoradiotherapy for Esophageal Squamous Cell Cancer: A Competing Risk Analysis of 358 Patients
Source: Cancer Med. 2026 Feb 8;15(2):e71610. doi: 10.1002/cam4.71610 (PMC12883298; doi:10.1002/cam4.71610)
Supplement: Supplementary file 1 — Table S1: Dosimetric parameters for heart and conduction nodes. [file CAM4-15-e71610-s001.docx]

**Supplemental Table 1 Dosimetric parameters for heart and conduction nodes**

| **Heart** | **Median** | **IQR** |
| --- | --- | --- |
| **Dmax,Gy** | 4444.05 | 4387.5-4513.6 |
| **Dmean,Gy** | 1545.05 | 1096.2-1871.4 |
| **V5, %** | 80.01 | 51.55-94.06 |
| **V10,%** | 58.84 | 39.87-71.67 |
| **V15,%** | 41.80 | 29.97-52.69 |
| **V20,%** | 29.94 | 20.09-38.15 |
| **V25,%** | 20.58 | 12.41-27.30 |
| **V30,%** | 13.44 | 8.2-19.5 |
| **V35,%** | 9.28 | 5.3-13.13 |
| **V40,%** | 5.17 | 2.9-7.77 |
| **SAN** |  |  |
| **Dmax,Gy** | 2617.4 | 1798.8-3413.2 |
| **Dmean,Gy** | 2003.35 | 1188.9-2610.8 |
| **V5, %** | 100 | 100-100 |
| **V10,%** | 100 | 69.04-100 |
| **V15,%** | 94.82 | 11.12-100 |
| **V20,%** | 50.2 | 0-99.52 |
| **V25,%** | 2.78 | 0-67.46 |
| **V30,%** | 0 | 0-19.3 |
| **V35,%** | 0 | 0-0 |
| **V40,%** | 0 | 0-0 |
| **AVN** |  |  |
| **Dmax,Gy** | 2749.3 | 1533.8-3580.4 |
| **Dmean,Gy** | 1845.65 | 458.7-2383.5 |
| **V5, %** | 100 | 32.27-100 |
| **V10,%** | 100 | 8.65-100 |
| **V15,%** | 75.93 | 0.07-100 |
| **V20,%** | 33.17 | 0-83.05 |
| **V25,%** | 2.56 | 0-39.46 |
| **V30,%** | 0 | 0-9.97 |
| **V35,%** | 0 | 0-0.18 |
| **V40,%** | 0 | 0-0 |
