# Supplementary material for: Whole Heart Dose Parameters Predict Severe Arrhythmias After Neoadjuvant Chemoradiotherapy for Esophageal Squamous Cell Cancer: A Competing Risk Analysis of 358 Patients
Source: Cancer Med. 2026 Feb 8;15(2):e71610. doi: 10.1002/cam4.71610 (PMC12883298; doi:10.1002/cam4.71610)
Supplement: Supplementary file 2 — Table S2: AUC value of 3‐year TimeROC result for arrhythmia. [file CAM4-15-e71610-s002.docx]

**Supplemental table 2: AUC value of 3-year TimeROC result for arrhythmia**

|  | **Characteristics** | **AF** | **Atrial flutter** | **Non-AF SVT** |
| --- | --- | --- | --- | --- |
| **Heart** | Dmax | 0.602 | 0.4178 | 0.6466 |
|  | Dmean | 0.592 | 0.5462 | 0.6456 |
|  | V5 | 0.5651 | 0.6424 | 0.6535 |
|  | V10 | 0.5658 | 0.5141 | 0.6018 |
|  | V15 | 0.5678 | 0.4746 | 0.5768 |
|  | V20 | 0.5780 | 0.5228 | 0.6195 |
|  | V25 | 0.5802 | 0.5433 | 0.6533 |
|  | V30 | 0.5843 | 0.5841 | 0.6779 |
|  | V35 | 0.5885 | 0.5826 | 0.6783 |
|  | V40 | 0.6107 | 0.5622 | 0.6704 |
| **SAN** | Dmax | 0.5318 | 0.4735 | 0.5955 |
|  | Dmean | 0.4930 | 0.5090 | 0.5698 |
|  | V5 | 0.4782 | 0.4241 | 0.5748 |
|  | V10 | 0.5366 | 0.4994 | 0.5310 |
|  | V15 | 0.5436 | 0.5766 | 0.5667 |
|  | V20 | 0.5253 | 0.5149 | 0.5673 |
|  | V25 | 0.4986 | 0.5493 | 0.5623 |
|  | V30 | 0.4924 | 0.4870 | 0.5807 |
|  | V35 | 0.4925 | 0.5249 | 0.5243 |
|  | V40 | 0.4694 | 0.4697 | 0.5928 |
| **AVN** | Dmax | 0.5133 | 0.5386 | 0.6533 |
|  | Dmean | 0.5248 | 0.5679 | 0.6606 |
|  | V5 | 0.4646 | 0.5821 | 0.5887 |
|  | V10 | 0.4982 | 0.55 | 0.6218 |
|  | V15 | 0.5108 | 0.5688 | 0.6603 |
|  | V20 | 0.5151 | 0.5876 | 0.6528 |
|  | V25 | 0.5590 | 0.5525 | 0.6678 |
|  | V30 | 0.5655 | 0.5049 | 0.6398 |
|  | V35 | 0.5196 | 0.5541 | 0.6101 |
|  | V40 | 0.5774 | 0.6174 | 0.5719 |
