# Supplementary material for: Whole Heart Dose Parameters Predict Severe Arrhythmias After Neoadjuvant Chemoradiotherapy for Esophageal Squamous Cell Cancer: A Competing Risk Analysis of 358 Patients
Source: Cancer Med. 2026 Feb 8;15(2):e71610. doi: 10.1002/cam4.71610 (PMC12883298; doi:10.1002/cam4.71610)
Supplement: Supplementary file 3 — Table S3: Competing risk regression models for Atrial Flutter. [file CAM4-15-e71610-s003.docx]

**Supplemental table 3 Competing risk regression models for Atrial Flutter**

| **Characteristics** | **Univariate** | | **Multivariate** | |
| --- | --- | --- | --- | --- |
|  | **HR (95%CI)** | **P value** | **sHR(95%CI)** | **P value** |
| **Age** |  |  |  |  |
| **≤65** | 1 |  |  |  |
| **>65** | 2.76(0.55-13.9) | 0.22 |  |  |
| **Sex** |  |  |  |  |
| **Female** | 1 |  |  |  |
| **Male** | NA |  |  |  |
| **BMI** |  |  |  |  |
| **<20** | 1 |  |  |  |
| **≥20** | 1.88(0.25-14.5) | 0.54 |  |  |
| **Baseline Hypertension** |  |  |  |  |
| **No** | 1 |  |  |  |
| **Yes** | 1.15(0.27-4.88) | 0.85 |  |  |
| **Diabetes** |  |  |  |  |
| **No** | 1 |  |  |  |
| **Yes** | 1.38(0.17-11) | 0.76 |  |  |
| **Baseline arrhythmia** |  |  |  |  |
| **No** | 1 |  |  |  |
| **Yes** | 1.18(0.25-5.63) | 0.84 |  |  |
| **Baseline CHD** |  |  |  |  |
| **No** | 1 |  | 1 |  |
| **Yes** | 4.35(0.90-21) | 0.067 | 6.57(1.47-29.4)^a^ | 0.014 |
| **Tumor length** | 1(0.79-1.27) | 0.99 |  |  |
| **nCRT Regimen** |  |  |  |  |
| **Without pembrolizumab** | 1 |  |  |  |
| **With pembrolizumab** | 4.82(0.024-1.77) | 0.15 |  |  |
| **Smoking status** |  |  |  |  |
| **No** | 1 |  |  |  |
| **Yes** | 4.05(0.52-31.9) | 0.18 |  |  |
| **Drinking status** |  |  |  |  |
| **No** | 1 |  |  |  |
| **Yes** | 4.02(0.51-31.7) | 0.19 |  |  |
| **RT dose** |  |  |  |  |
| **Heart V5, ml (≤91.63Gy vs >91.63Gy)** | 7.72(1.49-39.9) | 0.015 | 9.35(1.8-48.4)^a^ | 0.0077 |
| **Heart V30, ml (≤23.13% vs >23.13%)** | 4.08(1.01-16.5) | 0.049 |  |  |
| **Heart V35, ml (≤21.38% vs >21.38%)** | 4.58(0.56-37.4) | 0.16 |  |  |
| **AVN V20, ml (≤76.29% vs >76.29%)** | 2.79(0.71-11) | 0.14 |  |  |
| **AVN V40, ml (≤2.37% vs >2.37%)** | 2.4(0.30-19.5) | 0.41 |  |  |

Abbreviations: CHD,

^a^multivariate analysis with baseline CHD and V5;
