# Supplementary material for: Whole Heart Dose Parameters Predict Severe Arrhythmias After Neoadjuvant Chemoradiotherapy for Esophageal Squamous Cell Cancer: A Competing Risk Analysis of 358 Patients
Source: Cancer Med. 2026 Feb 8;15(2):e71610. doi: 10.1002/cam4.71610 (PMC12883298; doi:10.1002/cam4.71610)
Supplement: Supplementary file 4 — Table S4: Competing risk regression models for other supraventricular tachycardia. [file CAM4-15-e71610-s004.docx]

**Supplemental Table 4 Competing risk regression models for other supraventricular tachycardia**

| **Characteristics** | **Univariate** | | **Multivariate** | |
| --- | --- | --- | --- | --- |
|  | **HR (95%CI)** | **P value** | **sHR(95%CI)** | **P value** |
| **Age** |  |  |  |  |
| **≤65** | 1 |  |  |  |
| **>65** | 1.39(0.55-3.52) | 0.49 |  |  |
| **Sex** |  |  |  |  |
| **Female** | 1 |  |  |  |
| **Male** | 0.90(0.26-3.05) | 0.86 |  |  |
| **BMI** |  |  |  |  |
| **<20** | 1 |  |  |  |
| **≥20** | 0.93(0.31-2.79) | 0.9 |  |  |
| **Baseline Hypertension** |  |  |  |  |
| **No** | 1 |  |  |  |
| **Yes** | 1.45(0.58-3.63) | 0.42 |  |  |
| **Diabetes** |  |  |  |  |
| **No** | 1 |  |  |  |
| **Yes** | 1.14(0.27-4.8) | 0.86 |  |  |
| **Baseline arrhythmia** |  |  |  |  |
| **No** | 1 |  |  |  |
| **Yes** | 0.45(0.11-1.93) | 0.28 |  |  |
| **Baseline CHD** |  |  |  |  |
| **No** | 1 |  | 1 |  |
| **Yes** | 3.29(1.12-9.69) | 0.03 | 1.20(1.08-10.1)^a^ | 0.036 |
| **Tumor length** | 1.03(0.88-1.21) | 0.71 |  |  |
| **nCRT Regimen** |  |  |  |  |
| **Without pembrolizumab** | 1 |  |  |  |
| **With pembrolizumab** | 0.67(0.24-1.84) | 0.43 |  |  |
| **Smoking status** |  |  |  |  |
| **No** | 1 |  |  |  |
| **Yes** | 0.89(0.35-2.27) | 0.81 |  |  |
| **Drinking status** |  |  |  |  |
| **No** | 1 |  |  |  |
| **Yes** | 0.71(0.28-1.77) | 0.46 |  |  |
| **RT dose** |  |  |  |  |
| **Heart V30, ml (≤20.36% vs >20.36%)** | 5.02(2.03-12.4) | 0.00049 | 4.65(1.77-12.22)^a^ | 0.0019 |
| **Heart V35, ml (≤11.92% vs >11.92%)** | 5.47(1.97-15.2) | 0.0011 |  |  |
| **Heart V40, ml (≤7.16% vs >7.16%)** | 5.71(2.06-15.9) | 0.00083 |  |  |
| **AVN Dmean (≤15.17Gy vs >15.17Gy)** | 5.66(1.31-24.4) | 0.02 |  |  |
| **AVN V25, ml (≤0.19% vs >0.19%)** | 5.98(1.39-25.8) | 0.016 |  |  |

Abbreviations: CHD,

^a^multivariate analysis with baseline CHD and heart V40;
